# A pooled analysis of 3 large multicenter trials confirms a survival advantage for *NPM1* ^mut^ AML in MRD^neg^ remission after intensive induction

**DOI:** 10.1002/hem3.70198

**Published:** 2025-08-22

**Authors:** Konstanze Döhner, Hartmut Döhner, Daniela Späth, Silke Kapp‐Schwoerer, Amanda Gilkes, Ian Thomas, Sean Johnson, Nicola Potter, Yana Bevan, Jad Othman, Nigel H. Russell, Christoph Röllig, Christian Thiede, Martin Bornhäuser, Thomas Oellerich, Tressa Hood, Jenna Elder, Luis A. Carvajal, Jorge DiMartino, Richard Dillon

**Affiliations:** ^1^ Department of Internal Medicine III Ulm University Hospital Ulm Germany; ^2^ Department of Medical Genetics, Haematology and Pathology Cardiff University Cardiff UK; ^3^ Department of Medical & Molecular Genetics, School of Basic & Medical Biosciences King's College London London UK; ^4^ Centre for Clinical Haematology, Department of Haematology Nottingham University Hospital Nottingham UK; ^5^ Laborartory for Molecular Hematology University Hospital TU Dresden Dresden Germany; ^6^ Department of Hematology/Oncology Goethe University Frankfurt, University Hospital Frankfurt am Main Germany; ^7^ German Cancer Consortium (DKTK) Partner Site Frankfurt/Mainz Frankfurt am Main Germany; ^8^ Frankfurt Cancer Institute Goethe University Frankfurt am Main Germany; ^9^ Kronos Bio, Inc. San Mateo California USA; ^10^ PharPoint Research, Inc. Durham North Carolina USA

The nucleophosmin 1 (*NPM1*) gene, which is mutated in approximately 30% of newly diagnosed acute myeloid leukemia (AML) patients, is a useful target for molecular measurable residual disease (MRD) monitoring.^1^, ^2^ In addition to their relative homogeneity, *NPM1* mutations are ideal molecular MRD markers because they are true founder mutations and are retained at the time of relapse in most patients.^3^, ^4^ The European LeukemiaNet (ELN) MRD Working Party recommends quantitative polymerase chain reaction (qPCR) for molecular MRD analysis in AML with targetable mutations, such as the *NPM1* mutation, as well as *CBFB*::*MYH11*, *RUNX1*::*RUNX1T1*, and *PML*::*RARA* gene fusions, since the high expression of these mutations may allow for greater sensitivity.^2^ Numerous studies using reverse transcriptase‐mediated quantitative polymerase chain reaction (RT‐qPCR) have shown clinically meaningful and statistically robust improvements in survival associated with achieving MRD‐negative complete remission (CR).^5^, ^6^, ^7^, ^8^, ^9^, ^10^ These observed associations between MRD and survival supported inclusion of CR_MRD−_ as a response criterion in the 2017 ELN AML recommendations and inclusion of CR with partial (CRh_MRD−_) and incomplete (CRi_MRD−_) hematologic recovery in the 2022 update.^11^, ^12^

The present study further explored the value of MRD assessment by pooling and analyzing patient‐level data from three studies, conducted by the German/Austrian AML Study Group (AMLSG), the UK National Cancer Research Institute (NCRI), and the Study Alliance Leukemia (SAL), to evaluate the relationship of *NPM1*‐mutant (*NPM1*m) MRD negativity to relapse‐free survival (RFS) and overall survival (OS) across a range of RT‐qPCR normalized copy number (NCN) thresholds (≤0.01–≤1000 copies *NPM1*m/10^4^ *ABL1*) for MRD‐negativity in bone marrow (BM) and peripheral blood (PB). Further, this study investigated the prognostic value of MRD negativity in patients achieving CR, CRh, or CRi.

Deidentified data for 635 patients who achieved CR, CRh, or CRi and had RT‐qPCR MRD in BM and/or PB data at a single time point (within 42 days from the start of cycle 2 of intensive chemotherapy) were provided by the AMLSG for the AMLSG 09‐09 trial (*N* = 358),^9^ UK NCRI for the AML17 trial (*N* = 209),^7^ and the SAL for the AML2003 trial (*N* = 68).^6^ Only hematologic responses by the end of two cycles were considered in the analyses. Additional patient (Supporting Information S1: Table 1) and analysis details can be found in the supplement. Those who achieved CR (*N* = 417) were initially analyzed separately from those who achieved CRh (*N* = 17) or CRi (*N* = 201) and were subsequently combined for further analysis (Supporting Information S1: Figure 1).

Among the patients who achieved morphologic CR, 328 had MRD data available in BM and 311 in PB within 42 days of the start of chemotherapy cycle 2. Representative RFS and OS curves (NCN cutoff value ≤ 0.1) indicate poorer survival outcomes for MRD‐positive patients, as compared to MRD‐negative patients, whether patients had a CR or CRh/CRi (Figure 1). The forest plots (Figure 1E,F) illustrate that the effect on RFS and OS is driven by MRD negativity rather than by the type of hematologic remission (CR vs. CRh/CRi), and that the results from PB are more predictive of outcome compared to those from BM. This finding was consistent across all six NCN thresholds and both tissue types (Table 1 and Supporting Information S1: Table 2).

**Figure 1:**
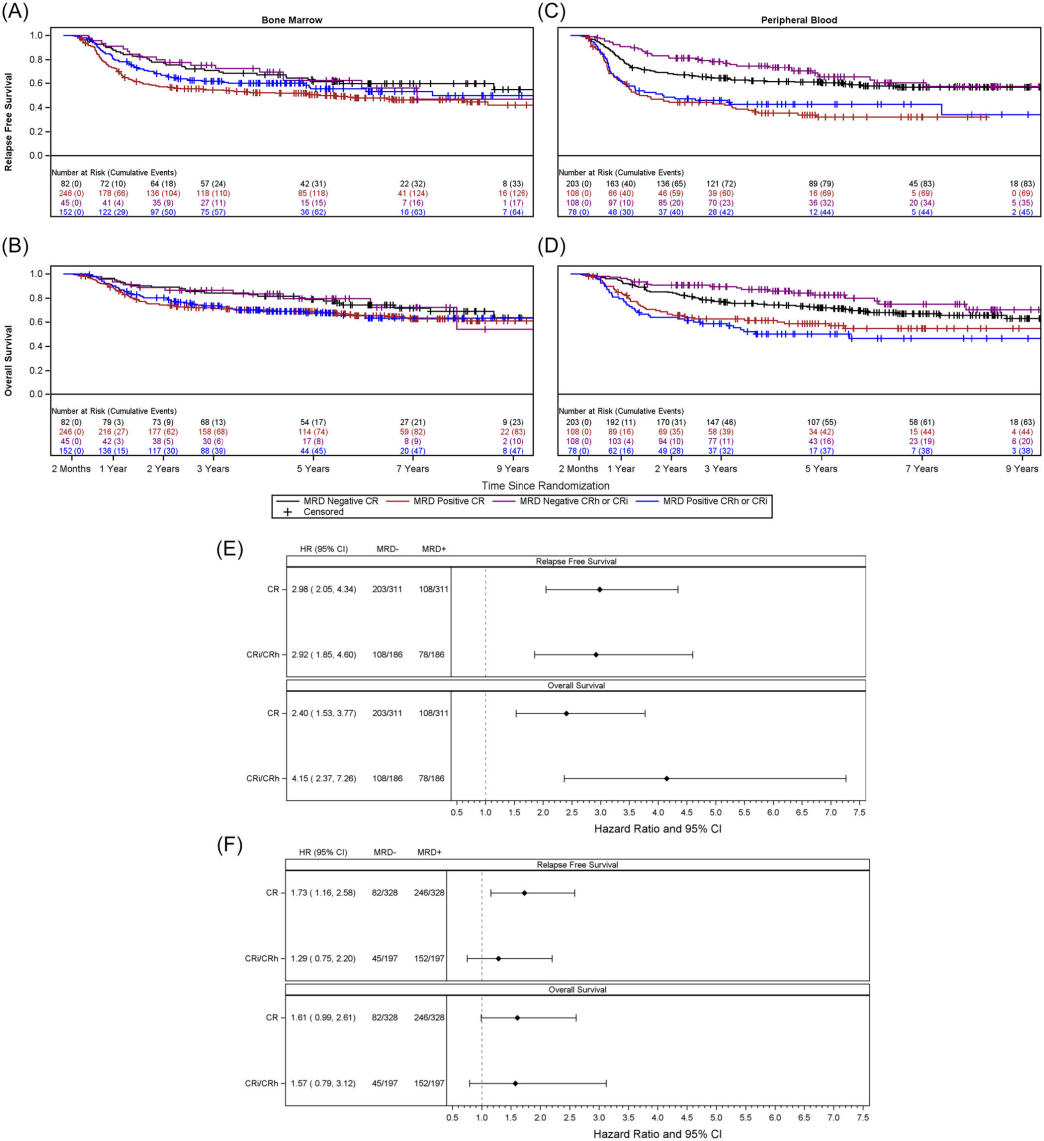
**Kaplan–Meier survival estimates in AML patients with CR, CRh, or CRi according to measurable residual disease (MRD) with the cut‐off** ≤ **0.1 (normalized copy number** ≤ **0.1** ***NPM1*** **m/10** ^**4**^ ***ABL1*** **)**. (**A**) Relapse‐free survival (RFS) of patients with MRD assessment of bone marrow (BM). (**B**) Overall survival (OS) of patients with MRD assessment of BM (**C**) RFS of patients with MRD assessment of peripheral blood (PB). (**D**) OS of patients with MRD assessment of PB. (**E**, **F**) Forest plots for hazard ratios for relapse‐free survival and overall survival comparing MRD positivity versus MRD negativity (cut‐off ≤ 0.1) in PB (**E**) and BM (**F**) by type of remission, CR versus CRi/CRh.

**Table 1:** Survival hazard ratios (HRs) for patients with MRD‐negative CR versus MRD‐positive CR, MRD‐negative CRh/CRi, and MRD‐positive CRh/CRi bone marrow and peripheral blood.

	Bone marrow, HR (95% CI) MRD‐negative CR versus			Peripheral blood, HR (95% CI) MRD‐negative CR versus		
MRD‐negative definition	MRD‐positive CR	MRD‐negative CRh/CRi	MRD‐positive CRh/CRi	MRD‐positive CR	MRD‐negative CRh/CRi	MRD‐positive CRh/CRi
Relapse‐free survival						
≤0.01 *NPM1*m/10^4^ *ABL1*	1.61 (1.06, 2.42)	1.02 (1.06, 2.42)	1.27 (0.79, 2.05)	2.97 (2.07, 4.27)	0.96 (0.62, 1.47)	2.76 (1.78, 4.28)
≤0.1 *NPM1*m/10^4^ *ABL1*	1.73 (1.16, 2.58)	1.05 (0.57, 1.94)	1.36 (0.85, 2.17)	2.98 (2.05, 4.34)	0.96 (0.63, 1.48)	2.82 (1.79, 4.42)
≤1 *NPM1*m/10^4^ *ABL1*	2.24 (1.55, 3.26)	1.21 (0.67, 2.17)	1.66 (1.06, 2.59)	3.19 (2.18, 4.65)	1.02 (0.68, 1.54)	2.74 (1.74, 4.32)
≤10 *NPM1*m/10^4^ *ABL1*	2.61 (1.79, 3.81)	1.32 (0.83, 2.09)	1.60 (1.03, 2.50)	2.88 (1.92, 4.31)	0.99 (0.69, 1.43)	2.55 (1.57, 4.15)
≤100 *NPM1*m/10^4^ *ABL1*	2.87 (1.92, 4.29)	1.05 (0.73, 1.50)	1.48 (0.90, 2.44)	3.28 (1.96, 5.50)	0.90 (0.65, 1.24)	3.23 (1.70, 6.16)
≤1000 *NPM1*m/10^4^ *ABL1*	3.84 (2.19, 6.74)	0.86 (0.63, 1.18)	1.88 (0.82, 4.30)	4.90 (2.35, 10.21)	0.86 (0.63, 1.16)	17.20 (6.61, 44.77)
Overall survival						
≤0.01 *NPM1*m/10^4^ *ABL1*	1.48 (0.90, 2.44)	0.97 (0.45, 2.11)	1.49 (0.84, 2.64)	2.56 (1.66, 3.95)	0.83 (0.48, 1.44)	3.54 (2.13, 5.89)
≤0.1 *NPM1*m/10^4^ *ABL1*	1.61 (0.99, 2.61)	1.01 (0.47, 2.19)	1.60 (0.91, 2.80)	2.40 (1.53, 3.77)	0.83 (0.48, 1.42)	3.44 (2.03, 5.82)
≤1 *NPM1*m/10^4^ *ABL1*	2.06 (1.31, 3.23)	1.15 (0.54, 2.42)	1.93 (1.13, 3.29)	2.49 (1.57, 3.94)	0.94 (0.57, 1.56)	3.22 (1.89. 5.46)
≤10 *NPM1*m/10^4^ *ABL1*	2.24 (1.42, 3.55)	1.34 (0.77, 2.36)	1.86 (1.09, 3.17)	2.56 (1.58, 4.17)	1.07 (0.69, 1.67)	3.14 (1.79, 5.50)
≤100 *NPM1*m/10^4^ *ABL1*	1.89 (1.13, 3.16)	1.07 (0.69, 1.65)	1.71 (0.95, 3.06)	2.95 (1.58, 5.52)	1.02 (0.69, 1.50)	4.50 (2.25, 9.02)
≤1000 *NPM1*m/10^4^ *ABL1*	3.89 (2.04, 7.41)	1.05 (0.72, 1.53)	2.53 (1.01, 6.32)	6.39 (2.87, 14.21)	1.02 (0.71, 1.47)	30.26 (11.20, 81.77)

*Note*: Complete remission (CR) was defined according to 2022 ELN criteria^12^ as bone marrow blasts <5%, absence of circulating blasts or blasts with Auer rods, absence of extramedullary disease, with an absolute neutrophil count (ANC) ≥ 1.0 × 10^9^/L and platelet count ≥ 100 × 10^9^/L; CR with partial hematologic recovery (CRh) required ANC ≥ 0.5 × 10^9^/L and platelet count ≥ 50 × 10^9^/L, otherwise all other CR criteria met; and CR with incomplete hematologic recovery (CRi) ANC < 1.0 × 10^9^/L or platelet count < 100 × 10^9^/L.

Abbreviations: CI, confidence interval; MRD, measurable residual disease.

Notably, there were differences in MRD negativity dependent upon whether the sample was acquired from BM or PB. A higher proportion of patients were MRD‐positive across all NCN thresholds when MRD was assessed from BM (Supporting Information S1: Figure 2).

To evaluate the predictive power of BM MRD positivity with respect to RFS, a receiver operating characteristic (ROC) analysis was conducted; at 36 months, BM and PB showed similar predictive power but revealed greater sensitivity for BM (*p* = 0.0017), suggesting that many patients with low levels of MRD detected in BM within 42 days of the start of cycle 2 of chemotherapy did not relapse (Supporting Information S1: Figure 3). Consistent with this, representative RFS and OS curves show larger separation between MRD‐positive and MRD‐negative patients for PB as compared to BM (Figure 1), as also shown across different cut‐of values (Supporting Information S1: Figure 4). These data are in line with the initial observations in the study by Ivey et al that the negative prognostic impact of MRD is greater for PB than for BM.^7^

Achievement of hematologic CR has traditionally served as a favorable response criterion in AML. Less stringent criteria were introduced, such as CRi and more recently CRh.^12^, ^13^ Studies have shown that patients with CRi may have inferior outcome to those with CR, but the association of CRh/CRi with outcome in the context of MRD has not been examined.^14^, ^15^ In this analysis, MRD positivity was associated with poorer RFS and OS in patients who achieved remission, regardless of whether peripheral count recovery was complete or incomplete (i.e., CRh/CRi) at the time of response assessment. Combining morphologic and MRD responses for analysis revealed that patients with MRD‐negative CRh/CRi show similar outcomes to patients with MRD‐negative CR (Figure 1 and Supporting Information S1: Table 2). These data suggest that, for *NPM1*m AML patients, achieving MRD negativity is of greater prognostic value than complete hematologic recovery at this early time point after two cycles of intensive chemotherapy.

Although CRh and CRi require the absence (<5%) of morphologic blasts, it has been hypothesized that lack of complete platelet and neutrophil recovery in these patients could be attributable to effects of residual leukemic burden on the BM microenvironment. Our analysis is inconsistent with this hypothesis since we could identify patients who had complete hematologic recovery despite the presence of detectable MRD and conversely, patients with partial or incomplete hematologic recovery who were MRD‐negative. In the context of the present study, patients with MRD‐positive CR actually displayed poorer RFS and OS as compared to patients with MRD‐negative CRh or CRi. The clinical value of responses such as CRh or CRi must be reevaluated to see whether achievement of MRD negativity may outperform the classic hematologic response criteria, particularly for clinical development of novel agents and chemotherapy combinations that may prevent timely and full hematologic recovery.

In alignment with previous studies, the analyses here suggest that capturing low levels of MRD in PB may offer the better prognostic value.^7^ Evidence suggests that in *NPM1*m AML MRD is more frequently detected at lower NCN thresholds in BM but may have less negative prognostic implications than similar levels of MRD detected in PB, so prospective studies evaluating predictive comparability between the two sample sources may be worthwhile. Sequential measurements of MRD, and the greater sensitivity offered by BM RT‐qPCR, could be most valuable to examine the kinetics of leukemic cell burden reduction or to monitor the kinetics of early relapse. It is worth noting that different sample preparations, e.g., using whole blood versus purified mononuclear cells for RNA extraction, could impact the signal‐to‐noise ratio and hence, the sensitivity of detection of mutant *NPM1*m transcripts. Overall, the results of our analysis provide further support for the use of post‐induction MRD assessment of mutant *NPM1* transcript level for earlier read‐out as well as a surrogate endpoint for outcome measures. Importantly, our data indicate that achievement of MRD negativity is of greater prognostic value than the achievement of full hematologic recovery, i.e., of a complete remission by ELN criteria. Our finding may be of value in revisiting the definition of treatment failure in event‐free survival analyses in the context of intensive chemotherapy that takes the type of hematologic response into account, that is, achieving CRh or CRi only being considered as an event, as currently proposed by the U.S. Food and Drug Administration AML Guidance Document.^16^ This may particularly become important in the context of upcoming randomized trials with targeted agents such as menin inhibitors in frontline therapy of *NPM1*m AML.

## AUTHOR CONTRIBUTIONS

**Konstanze Döhner**: Writing—original draft; conceptualization; investigation; methodology; validation; writing—review and editing; supervision; resources; project administration. **Hartmut Döhner**: Conceptualization; investigation; writing—original draft; methodology; validation; writing—review and editing; project administration; supervision; resources. **Daniela Späth**: Formal analysis; methodology; validation; data curation. **Silke Kapp‐Schwoerer**: Methodology; validation; investigation. **Amanda Gilkes**: Methodology; writing—review and editing; investigation. **Ian Thomas**: Investigation; methodology; writing—review and editing. **Sean Johnson**: Investigation; methodology; writing—review and editing. **Nicola Potter**: Investigation; methodology; writing—review and editing. **Yana Bevan**: Investigation; methodology; writing—review and editing. **Jad Othman**: Investigation; methodology; writing—review and editing. **Nigel H. Russell**: Conceptualization; investigation; writing—original draft; methodology; validation; writing—review and editing; supervision; resources. **Christoph Röllig**: Investigation; methodology; writing—review and editing. **Christian Thiede**: Investigation; methodology; writing—review and editing. **Martin Bornhäuser**: Investigation; methodology; writing—review and editing. **Thomas Oellerich**: Investigation; methodology; writing—review and editing. **Tressa Hood**: Software; formal analysis; data curation. **Jenna Elder**: Software; formal analysis; data curation. **Luis A. Carvajal**: Investigation; methodology; writing—review and editing. **Jorge DiMartino**: Conceptualization; investigation; writing—original draft; methodology; validation; writing—review and editing; supervision. **Richard Dillon**: Conceptualization; investigation; writing—original draft; methodology; validation; writing—review and editing; supervision.

## CONFLICT OF INTEREST STATEMENT

Konstanze Döhner: Consultancy with honoraria: AbbVie, Janssen, Jazz, Novartis, Bristol Myers Squibb, Celgene; Clinical research funding to institution: Novartis, AbbVie, Astellas, Bristol Myers Squibb, Celgene, Jazz Pharmaceuticals, Kronos Bio, Servier. Hartmut Döhner: Consultancy with honoraria: AbbVie, AstraZeneca, Gilead, Janssen, Jazz, Pfizer, Servier, Stemline, Syndax; Clinical research funding to institution: AbbVie, Astellas, Bristol Myers Squibb, Celgene, Jazz Pharmaceuticals, Kronos Bio, Servier. Daniela Späth: No conflicts of interest. Silke Kapp‐Schwoerer: Consultancy with honoraria: AbbVie, BMS, Jazz Pharmaceuticals, Pfizer. Amanda Gilkes: No conflicts of interest. Ian Thomas: Consultancy with honoraria: Jazz, Novatis. Sean Johnson: No conflicts of interest. Nicola Potter: No conflicts of interest. Yana Bevan: No conflicts of interest. Jad Othman: Consultancy with honoraria: Astellas, Jazz. Nigel H. Russell: No conflicts of interest. Christoph Röllig: Advisory role with honoraria for AbbVie, Amgen, Astellas, BMS, Celgene, Jazz, Novartis, Pfizer, Servier; clinical research funding from AbbVie, Novartis, Pfizer. Christian Thiede: CEO and co‐owner of AgenDix GmbH. Martin Bornhäuser: Consultancy with honoraria: ActiTrexx, Alexion, Jazz Pharmaceuticals, MSD. Thomas Oellerich: Consultancy with honoraria: AbbVie, BeiGene, Janssen, Kronos Bio, Merck KGaA, Roche; Research funding: Merck KGaA, Gilead; all not related to this publication. Tressa Hood: Kronos Bio, Inc. Jenna Elder: PharPoint Research, Inc. Luis A. Carvajal: Kronos Bio, Inc. Jorge DiMartino: Kronos Bio, Inc. Richard Dillon: Consultancy with honoraria: AbbVie, Astellas, Jazz, Pfizer, Servier, and membership of a Data Safety and Monitoring Board with AvenCell, Research support from AbbVie, Amgen, Jazz and Pfizer.

## FUNDING

K. Döhner and H. Döhner are supported by the Sonderforschungsbereich SFB 1074 project B3 and Z02, titled “Experimental models and clinical translation in leukemia”, funded by the Deutsche Forschungsgemeinschaft. Open Access funding enabled and organized by Projekt DEAL.

## Supporting information

Supporting Information.

## Data Availability

The data that support the findings of this study are available from the corresponding author upon reasonable request.
