# Supplementary material for: A pooled analysis of 3 large multicenter trials confirms a survival advantage for NPM1 mut AML in MRDneg remission after intensive induction
Source: Hemasphere. 2025 Aug 22;9(8):e70198. doi: 10.1002/hem3.70198 (PMC12371262; doi:10.1002/hem3.70198)

**Supplemental Appendix**

**A Pooled Analysis of 3 Large Multicenter Trials Confirms a Survival Advantage for *NPM1*^mut^ AML in MRD^neg^ Remission After Intensive Induction**

**Patient selection and assessment**

Deidentified patient data included demographic information, disease history, induction and consolidation treatments received, morphologic response, RT-qPCR MRD quantitation after 2 chemotherapy cycles, relapse, and survival status. These data were standardized using Standard Data Tabulation Models.

With regard to the induction regimens, we refer to the original publications of the clinical trials: AMLSG 09-09^1^; NCRI AML17^2^; SAL AML2003^3^.

Morphologic response was as assessed following induction per the guidelines of individual study. CR included absolute neutrophil count and platelet count recovery in accordance with the 2017 ELN recommendations.^4^

**MRD assays**

RT-qPCR was performed as previously described for the NCRI patients^5^ and for the AMLSG^6^ and SAL patients.^7^ RNA was extracted from whole blood or bone marrow for the NCRI patients while mononuclear cells were isolated from blood or bone marrow prior to RNA extraction for the AMLSG and SAL patients. For standardization of this analysis, RT-qPCR results from all of the studies were defined as *NPM1*m transcript copies per 10e^4^ *ABL* transcripts (normalized copy number [NCN]).

*RT-qPCR assay for AMLSG 09-09 study patients:* For quantification of *NPM1*^mut^ ribonucleic acid (RNA) we applied and modified the quantitative reverse transcription polymerase chain reaction (RQ-PCR) assay designed by Gorello et al.^8^ as previously described. We used *Abelson 1* (*ABL1*) as control gene.^9^ Real-Time Quantitative Polymerase Chain Reaction Analyses RQ-PCR assays were performed on the Applied Biosystems platform 7900 HT (Applied Biosystems, Foster City, CA) and QuantStudioTM 12K Flex Real-Time PCR System (Applied Biosystems, Foster City, CA). All reagents were derived from the TaqMan® PCR Core Reagents Kit (Applied Biosystems, Foster City, CA; since 2014 Life Sciences Solutions - Thermo Fisher Scientific, Darmstadt, DE). The 25μl reaction mix for NPM1mut quantification contained 3μl cDNA, 1.25U AmpliTaq Gold®, 0.25U AmpErase® UNG, 2.5μl 10x Taqman PCR buffer, 4mM MgCl, 500μM of each dNTP, 400nM of *NPM1* common forward primer (cNPM-C-F), 300nM *NPM1*^mut^ specific reverse primer, 200nM FAM/ MGB labeled *NPM1* probe. LNA was added to improve specificity for mutation types Nm and Km (0.125µl). The 25μl reaction mix for *ABL1* quantification contained 3μl cDNA, 1.25U AmpliTaq Gold®, 0.25U AmpErase® UNG, 2.5μl 10x Taqman PCR buffer, 4mM MgCl, 500μM of each dNTP, 300nM *ABL1* forward primer, 300nM *ABL1* reverse primer, and 200nM HEX/ MGB labeled *ABL1* probe. PCR conditions were 2 min at 50°C, 10 min polymerase activation at 95°C and 50 cycles of denaturation at 95°C for 15 sec and annealing at 62°C for 1 min. Each RQ-PCR run included a standard curve of a serial dilution from 106 to 101 plasmids for *ABL* and the respective *NPM1*^mut^ type, negative controls defined as no template controls (NTC), and *NPM1* wild-type patient sample or cell line. *NPM1*^mut^ and *ABL1* were amplified S-1 separately in three wells each. SDS 2.3 software and QuantStudioTM 12K Flex Software v1.1.1 (Applied Biosystems, Foster City, CA) were used to visualize amplification curves and calculate Ct values at a threshold of 0.1 and baseline set at 3-15 for *NPM1*^mut^ and *ABL1*. Prerequisites for evaluation were negativity in all NTCs and wild-type samples and a correlation coefficient of the standard curve ≥ 0.99. Primer and Probes for RQ-PCR mutation types were specified according to the nomenclature of Falini^10^. For MRD assessment of samples from patients with type D and Jt mutations the *NPM1*^mut^ type A assay was applicable. For the rare mutation types B, C, Km, Qm, Nm, and 4 we designed individual reverse primers. The sensitivity of our assay was 10^-5^ (type 4) up to 10^-6^ (types A, B, C, D, Jt, Qm, Nm, Km). Determination of specificity and sensitivity Sensitivities were determined by serial dilution of the cell line OCI-AML3 (*NPM1*^mut^ type A) or mutation-specific patient samples in HL60 (*NPM1*WT) cells. Maximum sensitivity was 10^-6^ for mutation types A, B, C, D, Jt, Qm, Km, Nm and 10^-5^ for mutations type 4. The assays were highly specific as Ct values in *NPM1*wt AML samples or *NPM1*wt cell lines were out of the quantitative range and no wildtype *NPM1* was detected. Definition of MRD positivity and MRD negativity: A sample was considered positive if the Ct was lower than the Y-intercept plus 1 in 2 of 3 wells. If only 1 of 3 wells was positive the test was repeated. For fulfilling the criteria “MRDneg” all three *NPM1*mut Ct values needed to be out of the quantitative range.

*RT-qPCR assay for NCRI AML17 study patients:* MRD testing for the UK NCRI was performed at the Cancer Genetics laboratory, Department of Medical and Molecular Genetics, Kings College London. An in-house mutation-specific RT-qPCR assay was used, based on the method of Krönke and colleagues^11^. RNA was isolated from lysed whole bone marrow aspirate or peripheral blood samples using Trizol reagent (Life Technologies, Carlsbad, CA) or Buffer RLT (Qiagen) and reverse transcribed using either ThermoScript or SuperScript III Reverse Transcriptase (both ThermoFisher). Mutation-specific reverse primers with a common forward primer and probe were used. Assays were run on an ABI 7900 or QuantStudio6 RT-PCR system (both ThermoFisher). *ABL1* was used as a control gene for all assays. Assays were performed under PCR conditions proposed by the Europe Against Cancer (EAC) program^9^ except for the annealing temperature, which was adjusted to eliminate background amplification. The detection threshold was manually set to 0.1 for both *NPM1* and *ABL1*. For non-ABD mutations, we used Primer Express software (Applied Biosystems) to design three separate mutation specific reverse primers, retaining the usual forward primer and probe. These primers were first tested on the patient’s diagnostic sample, and only primers which amplified *NPM1* mutated transcripts at the same or a lower cycle threshold than *ABL1* transcripts were accepted. These primers were then tested on a panel of known normal samples and only primers with no amplification in the normal samples were accepted. In some cases, this required adjustment of the annealing temperature. If more than one of the three tested primers fulfilled these criteria, the primer with the greatest difference between *ABL1* and *NPM1* cycle thresholds was chosen. Using this strategy, we could develop an assay for all mutations encountered in this study. Samples were run in triplicate. The definition of MRD-positivity was detection of amplification of *NPM1* mutated transcripts before PCR cycle 40 (i.e. cycle threshold, Ct < 40) in at least 2 of 3 triplicates. Samples with inadequate input RNA (*ABL1* Ct > 30) were excluded. Plasmid standards (Qiagen) were used to calculate copy number. The difference in cycle threshold method was used to estimate copy numbers for non-ABD transcript types where plasmid standards were not available.

*RT-qPCR assay for SAL AML2003 study patients:* Initial screening for *NPM1* mutations was performed by polymerase chain reaction (PCR) on genomic DNA using either the published primer molecules *NPM1*-F and *NPM1*-R or primers NPM-I11f (5-CTGGTAGAATGAAAAATAGAT-3) and NPM-E12r (5 -CTTGGCAATAGAACCTGGAC-3). Primers NPM1-F and NPM-I11f were labeled with 6-FAM or Hex (TIB MolBiol, Berlin, Germany). All samples that were positive for mutant *NPM1* were then sequenced by using a locked nucleic acid (LNA) clamping strategy. For MRD quantiﬁcation, an improved qPCR assay for the detection of the three most common *NPM1* mutations (types A, B, and D) was developed. By using published assays, a high rate of unspeciﬁc ampliﬁcation was observed for the most common variant (mutation type A; duplication of the bases TCTG). To overcome this unspeciﬁc ampliﬁcation, LNA bases were incorporated into the primer sequences of the mutant-speciﬁc primer. A common generic probe and reverse primer were used for all reactions. PCR was performed on a LightCycler480 instrument (Roche, Mannheim, Germany) using 5’-nuclease chemistry. In the ﬁnal optimized procedure, the 20-µL reaction mixture consisted of 4 µL of LightCycler TaqMan Master kit (Roche), 0.25 mM *NPM1* mutation–speciﬁc primer, 0.3 mM common NPMs primer, 0.2 mM generic NPM1 probe, 12.5 mL of water, and up to 2 µL of complementary DNA (cDNA), prepared from 1 to 5 mg of total RNA. After an initial step of 95°C for 10 minutes, 50 cycles of a two-step PCR were performed, with 20 seconds at 94°C for denaturation and 1 minute at 66°C for annealing and elongation. Absolute quantiﬁcation was performed based on standard curves by using cloned cDNAs of the individual mutants. Results were adjusted to *ABL1* as the reference gene and expressed as percent *NPM1*^mut^ /*ABL1*. A minimum *ABL1* copy number of 1000 copies was required for inclusion of a sample; the median *ABL1* copy number of all samples investigated was 30 000. All samples without a signal after 50 cycles were considered negative for *NPM1*^mut^. A reaction containing MV4-11 (wild-type NPM1 [wt-NPM1]) cells was run with every qPCR to control for unspeciﬁc ampliﬁcation, along with appropriate positive and no-template controls.

**Statistical methods**

OS was assessed from time of randomization to death. For patients with no evidence of death, OS was censored at the last follow-up where the patient was confirmed alive. For patients with CR, RFS was defined as the time from date of achievement of remission until the date of hematologic relapse or death from any cause; patients without any known relapse or death were censored at their last study visit.^13^ Morphologic response and MRD assessments had to be completed within 42 days of the start of Cycle 2 to be included in the analysis as per FDA guidance.

**Receiver operating characteristic (ROC) analysis**

Time-dependent receiver operating characteristic (ROC) curves were developed for patients with any CR, to determine the appropriate cutoff for MRD using peripheral blood or bone marrow samples. For this analysis, NCN was categorized into 7 groups ranging from ≤ 0.01 *NPM1*m transcripts/10^4^ *ABL1* (≤ 0.01 NCN) to >1000 NCN. RFS was modeled separately for BM and PB with time-dependent Cox Regression models using the categorized NCN as the dependent variable. The area under the curve (AUC) at 36 months were calculated. Using patients who were alive at month 36 with paired samples BM and PB, the agreement of the assays utilizing the optimal cutoffs was assessed using a McNemar’s test.

**References**

1. Döhner H, Weber D, Krzykalla J, et al; German–Austrian AML Study Group. Intensive chemotherapy with or without gemtuzumab ozogamicin in patients with NPM1-mutated acute myeloid leukaemia (AMLSG 09-09): a randomised, open-label, multicentre, phase 3 trial. *Lancet Haematol*. 2023;10(7):e495-e509.

2. Burnett AK, Russell NH, Hills RK, et al; UK NCRI AML Study Group. A randomized comparison of daunorubicin 90 mg/m2 vs 60 mg/m2 in AML induction: results from the UK NCRI AML17 trial in 1206 patients. *Blood*. 2015;125(25):3878-85.

3. Schaich M, Parmentier S, Kramer M, et al. High-dose cytarabine consolidation with or without additional amsacrine and mitoxantrone in acute myeloid leukemia: results of the prospective randomized AML2003 trial. *J Clin Oncol*. 2013;31(17):2094-102.

4. Döhner H, Estey E, Grimwade D, et al. Diagnosis and management of AML in adults: 2017 ELN recommendations from an international expert panel. Blood. 2017;129(4):424-447.

5. Ivey A, Hills RK, Simpson MA, et al. Assessment of Minimal Residual Disease in Standard-Risk AML. *N Engl J Med*. 2016;374(5):422-433.

1. Kapp-Schwoerer S, Weber D, Corbacioglu A, et al. Impact of gemtuzumab ozogamicin on MRD and relapse risk in patients with NPM1-mutated AML: results from the AMLSG 09-09 trial. Blood. 2020;136(26):3041-3050.
2. Shayegi N, Kramer M, Bornhauser M, et al. The level of residual disease based on mutant NPM1 is an independent prognostic factor for relapse and survival in AML. Blood. 2013;122(1):83-92.
3. Gorello P, Cazzaniga G, Alberti F, et al. Quantitative assessment of minimal residual disease in acute myeloid leukemia carrying nucleophosmin (NPM1) gene mutations. Leukemia. 2006;20(6):1103-8.
4. Gabert J, Beillard E, van der Velden VH, et al. Standardization and quality control studies of 'real-time' quantitative reverse transcriptase polymerase chain reaction of fusion gene transcripts for residual disease detection in leukemia - a Europe Against Cancer program. Leukemia. 2003;17(12):2318-57.
5. Falini B, Nicoletti I, Martelli MF, et al. Acute myeloid leukemia carrying cytoplasmic/mutated nucleophosmin (NPMc+ AML): biologic and clinical features. Blood. 2007;109(3):874-885.
6. Krönke J, Schlenk RF, Jensen KO, Tschürtz F, Corbacioglu A, Gaidzik VI, Paschka P, Onken S, Eiwen K, Habdank M, Späth D, Lübbert M, Wattad M, KindlerT, Salih HR, Held G, Nachbaur D, von Lilienfeld-Toal M, Germing U, Haase D, Mergenthaler HG, Krauter J, Ganser A, Göhring G, Schlegelberger B, Döhner H, Döhner K: Monitoring of Minimal Residual Disease in *NPM1* Mutated Acute Myeloid Leukemia: A study of the German-Austrian AML Study Group (AMLSG). J Clin Oncol. 2011;29:2709-16.
7. Döhner H, Wei AH, Appelbaum FR, et al. Diagnosis and management of AML in adults: 2022 recommendations from an international expert panel on behalf of the ELN. Blood. 2022;140(12):1345-1377.

**Supplemental Table 1.** **Patient demographics of study patients and patients in CR, CRh, CRi without MRD data**

|  | **AMLSG 09-09 (N=358)** | **NCRI AML17 (N=209)** | **SAL AML2003 (N=68)** | **Study patients (N=635)** | **Patients in CR, CRh, CRi without MRD data (N=140)** | |
| --- | --- | --- | --- | --- | --- | --- |
| **Age (years)** | | | | |  | |
| <60 | 212 (59.2%) | 168 (80.4%) | 64 (94.1%) | 444 (69.9%) | 92 (65.7%) | |
| ≥60 | 146 (40.8%) | 41 (19.6%) | 4 (5.9%) | 191 (30.1%) | 48 (34.3%) | |
| **Sex** | | | | |  | |
| Male | 166 (46.4%) | 98 (46.9%) | 26 (38.2%) | 290 (45.7%) | 66 (47.1%) | |
| Female | 192 (53.6%) | 111 (53.1%) | 42 (61.8%) | 345 (54.3%) | 74 (52.9%) | |
| **Race** | | | | |  | |
| Asian | 1 (0.3%) | 4 (1.9%) | 0 | 5 (0.8%) | 4 (2.9%) | |
| Black or African American | 0 | 5 (2.4%) | 0 | 5 (0.8%) | 1 (0.7%) | |
| White | 347 (96.9%) | 192 (91.9%) | 0 | 539 (84.9%) | 131 (93.6%) | |
| Other | 10 (2.8%) | 7 (3.3%) | 0 | 17 (2.7%) | 2 (1.4%) | |
| Not reported | 0 | 1 (0.5%) | 68 (100.0%) | 69 (10.9%) | 2 (1.4%) | |
| **AML subtype** | | | | |  | |
| De novo | 334 (93.3%) | 200 (95.7%) | 66 (97.1%) | 600 (94.5%) | 136 (97.1%) | |
| Treatment related | 24 (6.7%) | 9 (4.3%) | 2 (2.9%) | 35 (5.5%) | 4 (2.9%) | |
| Secondary | 0 | 0 | 0 | 0 | 0 | |
| **ECOG/WHO PS** | | | | |  | |
| 0 | 130 (36.3%) | 136 (65.1%) | 25 (36.8%) | 291 (45.8%) | 81 (57.9%) | |
| 1 | 192 (56.3%) | 62 (29.7%) | 32 (47.1%) | 286 (45.0%) | 46 (32.9%) | |
| 2 | 36 (10.1%) | 7 (3.3%) | 8 (11.8%) | 51 (8.0%) | 10 (7.1%) | |
| 3 | 0 | 4 (1.9%) | 0 | 4 (0.6%) | 3 (2.1%) | |
| Missing/Unknown | 0 | 0 | 3 (4.4%) | 3 (0.5%) | 0 | |
| **ELN 2022 Risk Category** | | | | |  | |
| Favorable | 308 ( 86.0%) | 131 ( 63.9%) | 48 ( 70.6%) | 487 ( 77.2%) | 93 (66.4%) | |
| Intermediate | 50 ( 14.0%) | 74 ( 36.1%) | 20 ( 29.4%) | 144 ( 22.8%) | 45 (32.1%) | |
| Missing/Unknown | 0 | 4 ( 1.9%) | 0 | 4 ( 0.6%) | 2 (1.4%) | |
| ***FLT3* Mutation** | | | | | | |
| Wildtype | 268 ( 74.9%) | 102 ( 49.8%) | 46 ( 67.6%) | 416 ( 65.9%) | 76 (54.3%) | |
| Mutation | 90 ( 25.1%) | 103 ( 50.2%) | 22 ( 32.4%) | 215 ( 34.1%) | 62 (44.3%) | |
| Missing/Unkonwn | 0 | 4 ( 1.9%) | 0 | 4 ( 0.6%) | 2 (1.4%) | |
| ***FLT3*** **Mutation Type^1^** | | | | | | |
| ITD | 50 ( 14.0%) | 74 ( 35.4%) | 20 ( 29.4%) | 144 ( 22.7%) | 45 (32.1%) | |
| TKD | 40 ( 11.2%) | 29 ( 13.9%) | 2 ( 2.9%) | 71 ( 11.2%) | 17 (12.1%) | |
| **Patients with AlloSCT** | | | | | | |
| AlloSCT received (yes)^2^ | 55 ( 15.4%) | 13 ( 6.2%) | 15 ( 22.1%) | 83 ( 13.1%) | NA | |
|  | | | | | |  |
| AML, acute myeloid leukemia; ECOG, Eastern Cooperative Oncology Group; ELN, European LeukemiaNet; NA, not available; PS, performance status; WHO, World Health Organization.  Note: Includes all patients with a CR, CRh, or CRi within 42 days forllowing the start of chemotherapy cycle 2 and have MRD from either assay in the same period.  ^1^ No patient with FLT3-mutated AML received a FLT3 inhibitor in frontline therapy  ^2^ Includes all patients in whom an AlloSCT was performed after the first instance of CR/CRi but prior to first instance of relapse, or information on relapse is missing. | | | | | | |

**Supplemental Table 2. 3-year (36-month) A) relapse-free survival (RFS) and B) overall survival (OS) for patients with MRD-negative CR vs. MRD-positive CR, MRD-negative CRh/CRi, and MRD-positive CRh/CRi bone marrow and peripheral blood.**


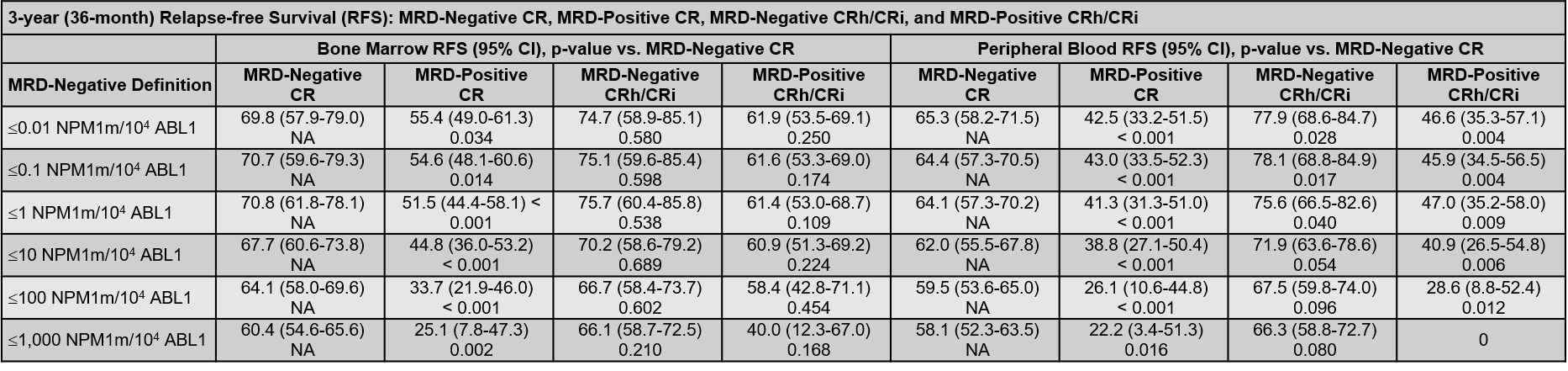
A Relapse-free survival


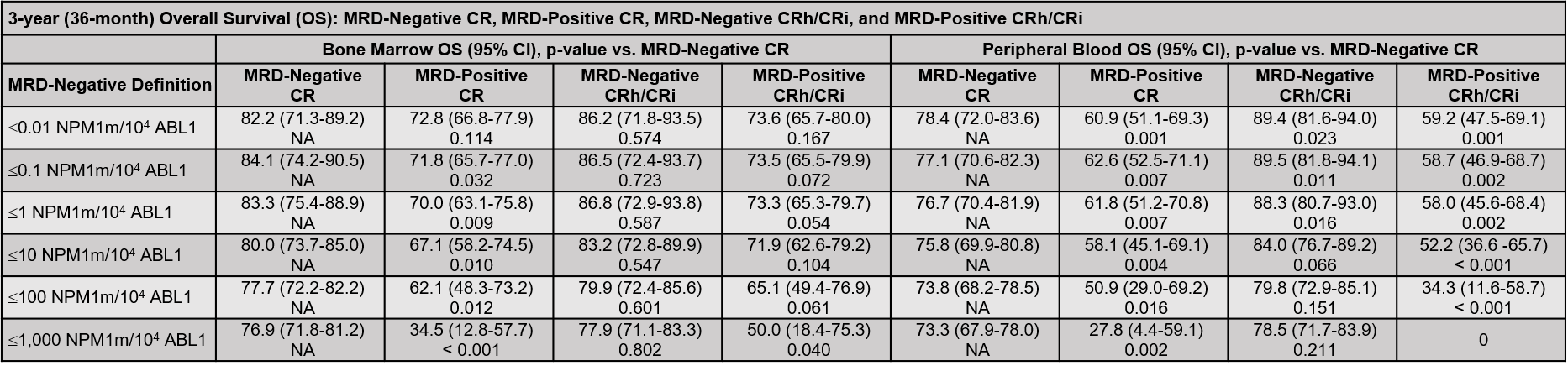
B Overall survival

**Supplemental Figure 1. Consort Diagram.** Derivation of study population from UK NCRI AML17, AMLSG 09-09 and AML2003 trials.


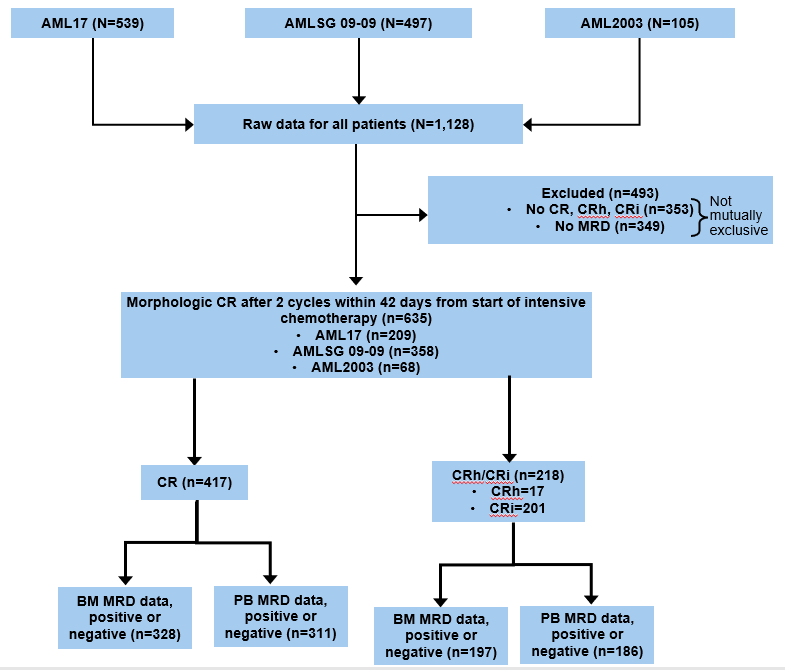


**Supplemental Figure 2. Number and proportion of patients in CR after two cycles of chemotherapy classified as MRD-negative or MRD-positive based on various copy number thresholds in bone marrow and peripheral blood.**

A Bone marrow


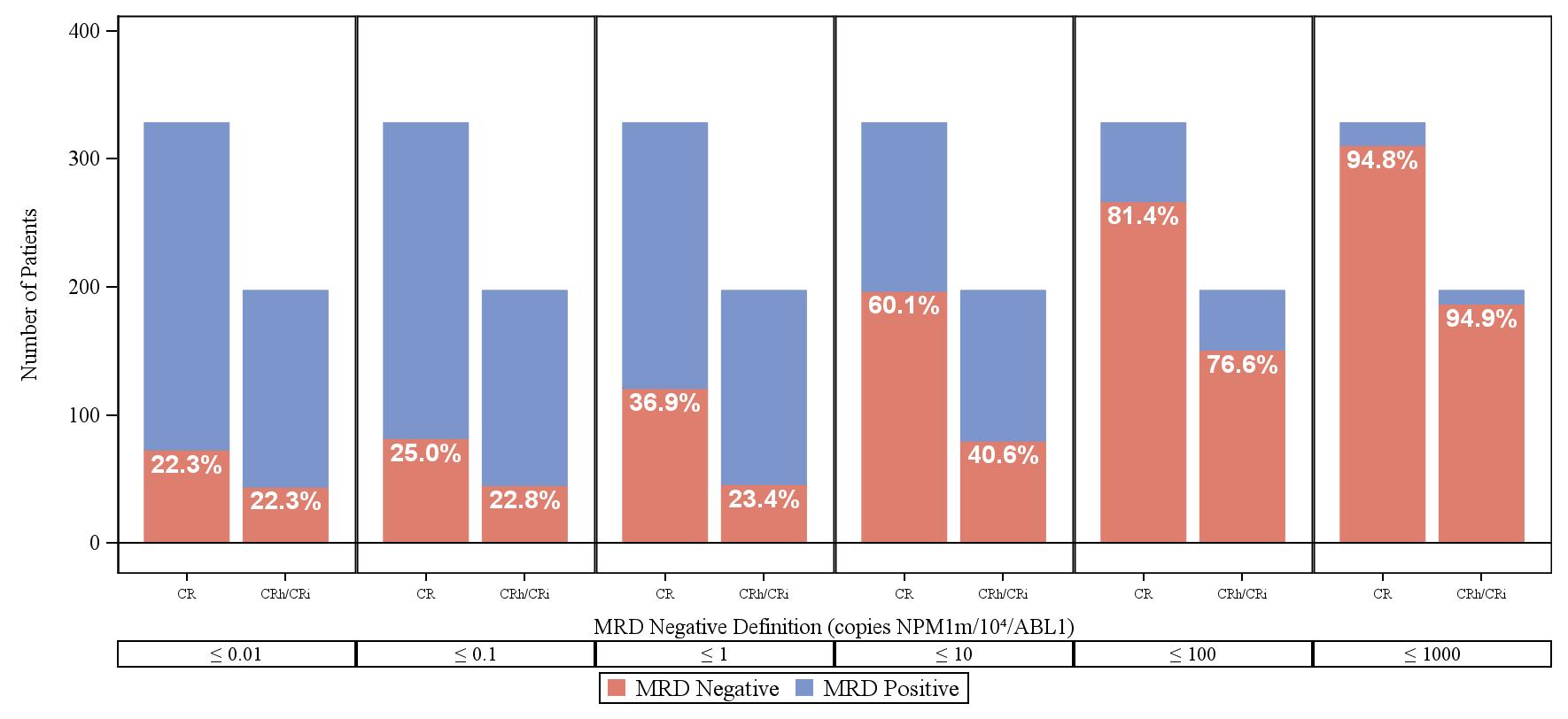


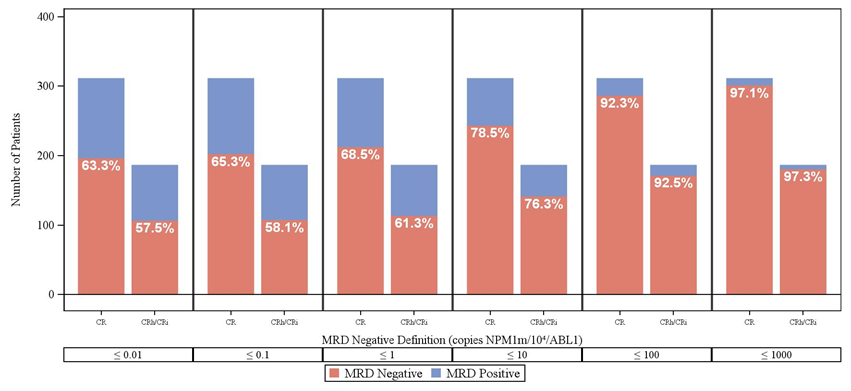
B Peripheral blood

**Supplemental Figure 3. Receiver operating characteristic (ROC) curves of relapse-free survival (RFS) for all patients with CR, CRh, or CRi from bone marrow and peripheral blood at 36 months.**

*Note*: The relatively poor sensitivities with RFS suggest that there are limitations to the MRD assays due to the possibility of false positive results.


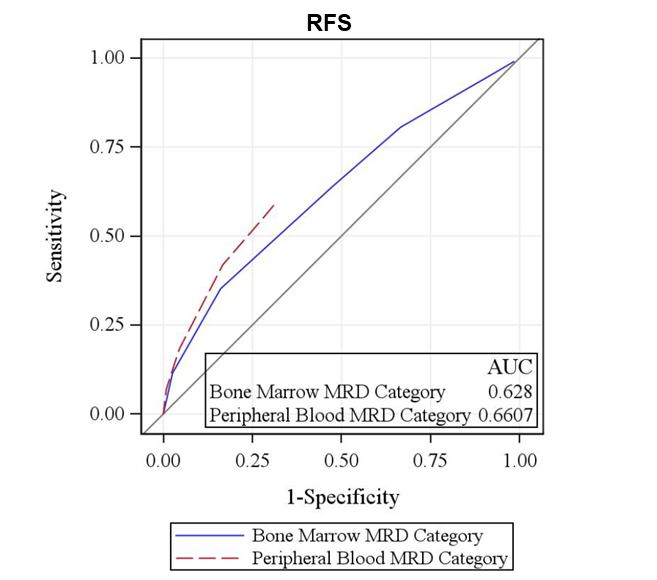


**Supplemental Figure 4: Relapse-free survival (RFS) and overall survival (OS) of patients with MRD negative *versus* MRD positive CR in bone marrow and peripheral blood with all cut-off levels ranging from ≤0.01 to 100 *NPM1*m/10^4^ *ABL1*.** Across all cut-off values, there is a better separation of the RFS and OS curves in peripheral blood *versus* bone marrow.

**A ≤0.01 *NPM1*m/10^4^ *ABL1***


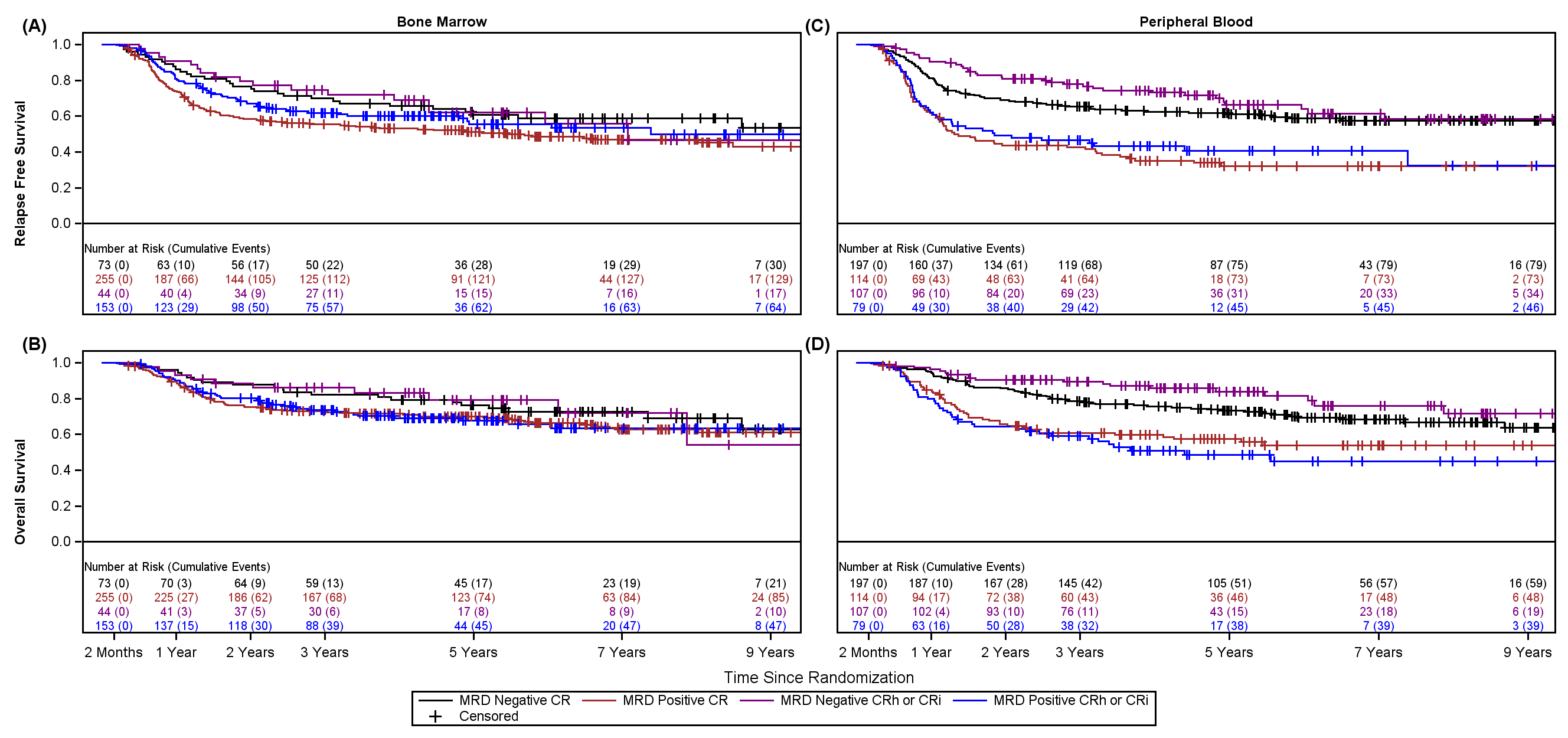


**B ≤0.1 *NPM1*m/10^4^ *ABL1***

**
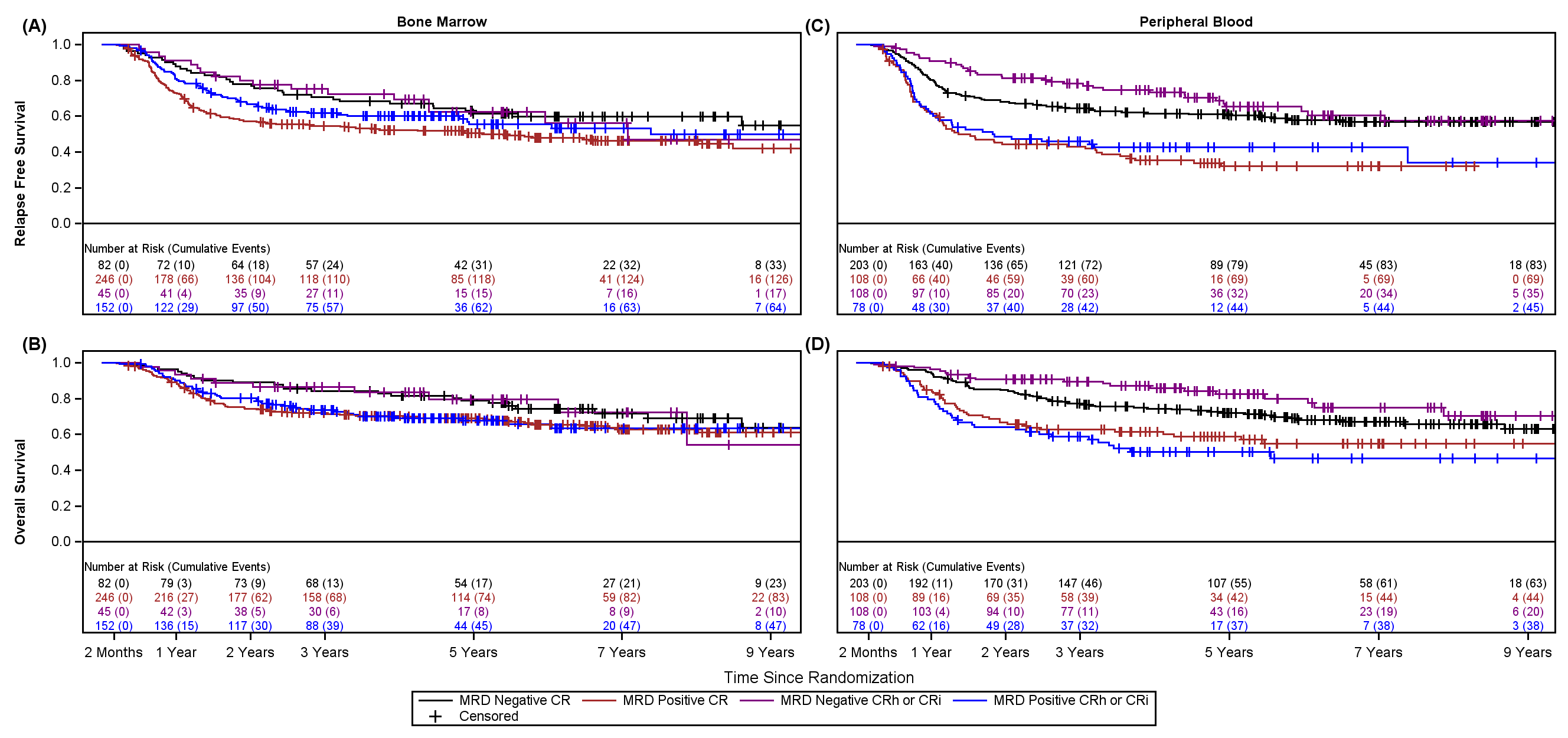
**

**C ≤1 *NPM1*m/10^4^ *ABL1***

***
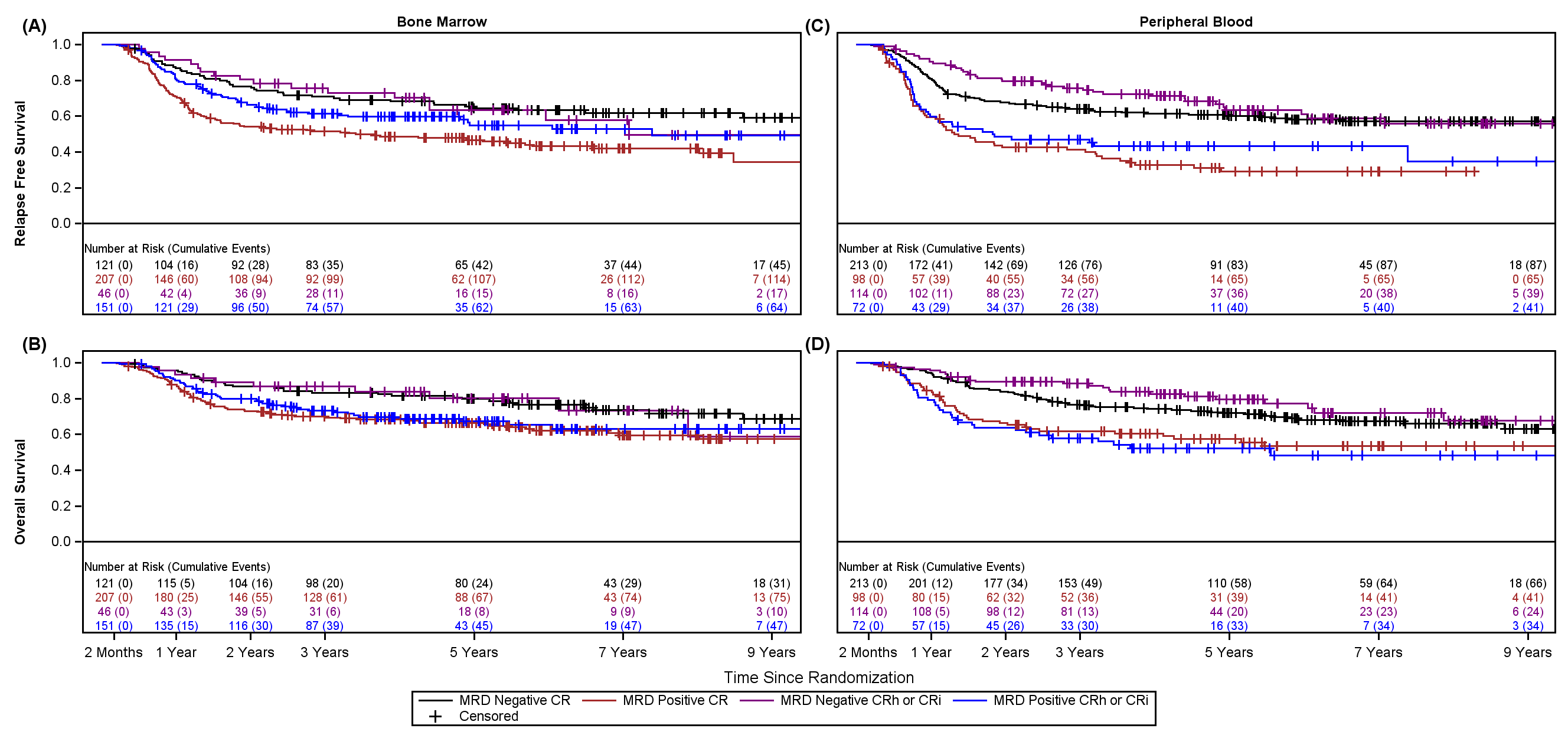
***

**D ≤10 *NPM1*m/10^4^ *ABL1***

**
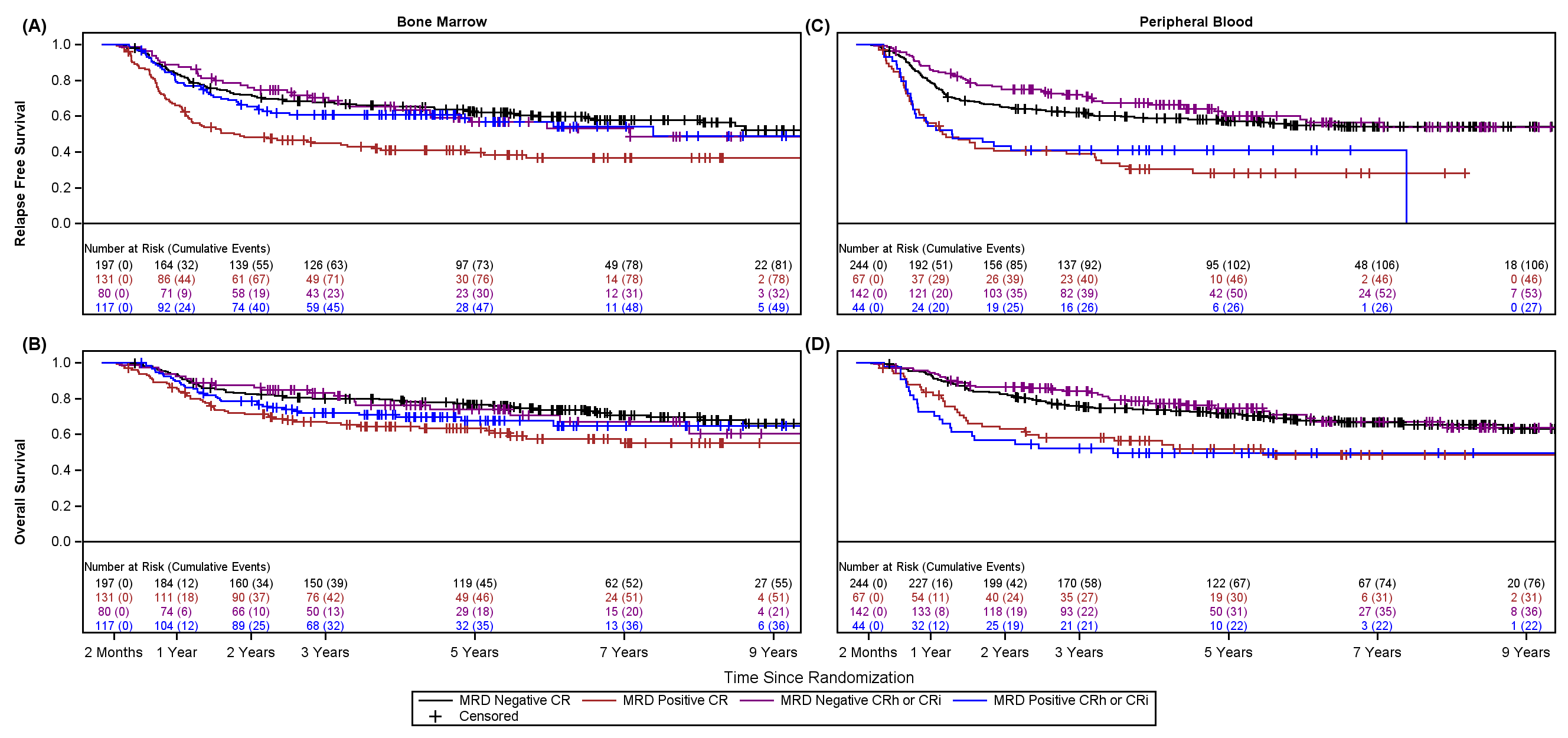
**

**E ≤100 *NPM1*m/10^4^ *ABL1***

**
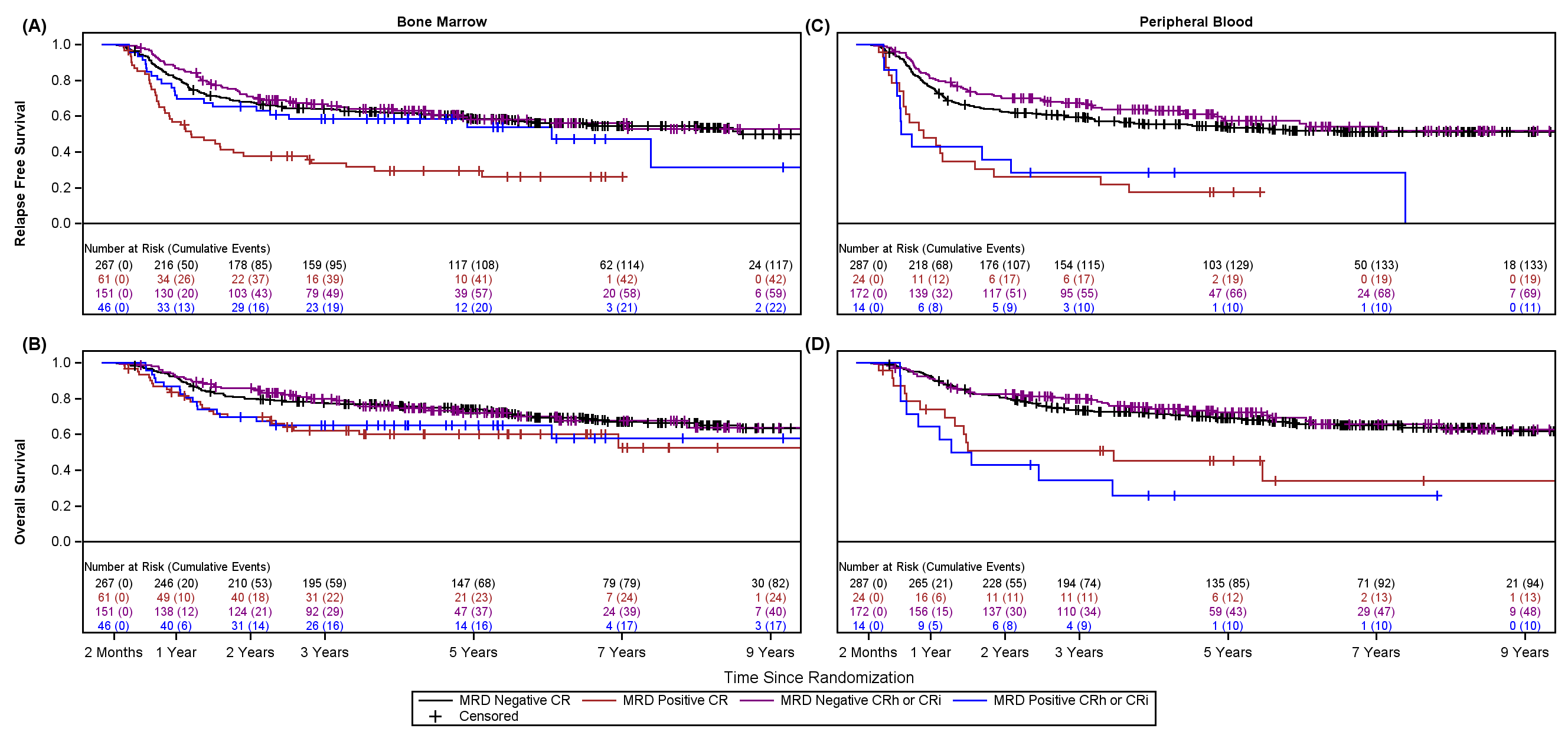
**

**Supplemental Figure 5: Overall survival of patients who achieved CR, CRh, CRi with MRD (n=635) data available *versus* patiens who achieved CR, CRh, CRi without MRD (n=140) data available within 42 days following the start of chemotherapy cycle 2.**

Note: Overall survival is defined as the time from randomization to death from any course. Patient who do not die will be censored at their last contact date.


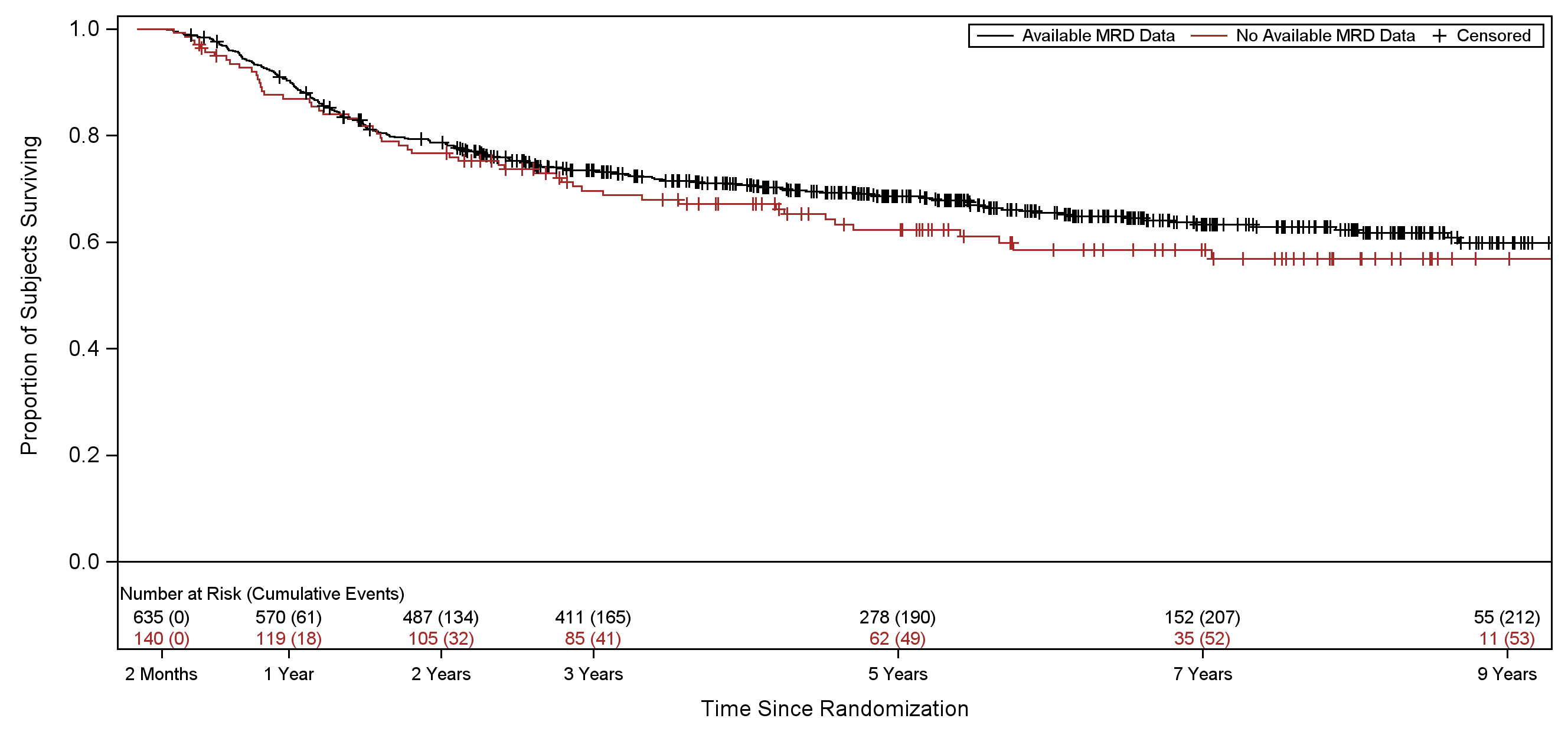

Supplement: Supplementary file 1 — Supporting Information. [file HEM3-9-e70198-s001.docx]
